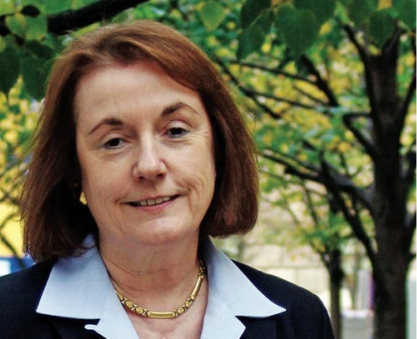# Kathryn R. Mahaffey: 1943–2009

**Published:** 2009-08

Kathryn R. Mahaffey, PhD, passed away 2 June 2009 after decades of work that advanced the nation’s health and environment. She is remembered as a source of inspiration with her principled and tireless intellect. She was the rare scientist who knew how to apply the lessons from academic research to protect the public heath. Her work changed the course of epidemic poisoning by heavy metals, endocrine disruptors, and many other environmental pollutants that particularly affect children, pregnant women, and disadvantaged populations. Mahaffey was the first to ensure that the number of lead-poisoned children in the United States was determined accurately through the National Health and Nutrition Examination Survey in the 1970s, an action that enabled the nation to track a reduction of more than 90% in children’s blood lead levels. Since then, as a result of her work, millions of children have avoided the tragedy of lead and mercury poisoning.

Mahaffey conducted pathbreaking research on mercury poisoning, helping to disentangle the web of bioaccumulation that had stymied previous efforts to seriously address the problem. She was a principal author of the 8-volume *Mercury Study Report to Congress*, which broke new scientific ground while focusing national attention on mercury exposure in the United States. Most recently, she helped organize an international conference in Japan that focused on methods to minimize exposure to mercury from eating contaminated fish while maximizing intake of key nutrients such as omega-3 polyunsaturated fatty acids. As a public health activist, her work won cheers from children’s health scientists and attacks from those who considered the facts she uncovered to be injurious to their interests.

Mahaffey graduated from The Pennsylvania State University and held a doctorate in nutrition, physiology, and biochemistry from Rutgers, The State University of New Jersey. She joined the public service in 1972, working first at the Food and Drug Administration, followed by positions at the National Institute for Occupational Safety and Health, the National Institute of Environmental Health Sciences, and the U.S. Environmental Protection Agency. Most recently she was a distinguished professorial lecturer at The George Washington University, where she taught toxicology. She was also engaged in helping to design new studies such as the National Children’s Study.

The recipient of numerous awards from government and academe, Mahaffey received the prestigious Arnold J. Lehman Award from the Society of Toxicology for her work in regulatory toxicology and the Bronze Medal for Commendable Service from the U.S. Environmental Protection Agency for her work on mercury. She was also appointed to many panels by the National Academy of Sciences. She most recently filed a scientific critique of a government report on risks and benefits of fish consumption; in her comments she demonstrated that an attempt to abandon fish advisories, which have helped reduce mercury exposure, was without scientific foundation.

Mahaffey was a prolific writer, having published more than 100 articles in the peer-reviewed scientific literature, 8 reports to Congress, 15 book chapters, and 7 books. She will be remembered by her colleagues as a passionate advocate for public health and the environment. *Environmental Health Perspectives* will miss her insightful reviews and sustained support of the journal.

## Figures and Tables

**Figure:**